# Effects of Meteorological Conditions on PM_2.5_ Concentrations in Nagasaki, Japan

**DOI:** 10.3390/ijerph120809089

**Published:** 2015-08-03

**Authors:** Jianhua Wang, Susumu Ogawa

**Affiliations:** Space Engineering and Planning Laboratory, Graduate school of Engineering, Nagasaki University, 1-14 Bunkyo-machi, Nagasaki 852-8521, Japan; E-Mail: jianhuagirl@gmail.com

**Keywords:** PM_2.5_, meteorological variables, correlation analysis, long-range transport

## Abstract

The fine particulate matter (PM_2.5_) problem has attracted much scientific and public attention, due to its effects on visibility, human health, and global climate. There are three factors that have important effect on PM_2.5_ mass concentration: domestic pollutant emission sources, external sources outside of the country, and the meteorological conditions. Nagasaki is a coastal prefecture located at the westernmost part of Japan, which is an ideal location to study pollutants from long range transport and correlation between PM_2.5_ and meteorological conditions. In this paper, PM_2.5_ concentration data and meteorological data were obtained during 1 January 2013~31 December 2013. The spatial distribution depicts that the western part of the study area has the most serious PM_2.5_ pollution. The correlation analysis results between PM_2.5_ concentration and meteorological data showed that temperature had a negative, and precipitation had a positive, correlation with PM_2.5_. There was a threshold in the correlations between humidity and wind speed and PM_2.5_. The correlation was positive or negative depending on the meteorological variable values, if these were lower or higher than the threshold. From the relationship with wind direction, it can be depicted that the west wind might bring the most pollutants to Nagasaki.

## 1. Introduction

With the development of economics in the world, the air pollution problem has become more and more serious in recent years, especially the fine particulate matter (PM_2.5_) problem [1,2,3]. PM_2.5_ is fine particle matter with an aerodynamic diameter less than 2.5 µm. Due to the effects on visibility, human health, and global climate, PM_2.5_ has attracted much scientific and public attention [4,5,6] Aerosol particles have been widely studied in the last ten years due to their potential health impact and demand for their control. Various studies have indicated that fine aerosol particles have the strongest health effects [7]. In addition, aerosol particles are of great importance in affecting atmospheric radiation, cloud formation, atmospheric photochemical reactions, and light extinction effects that influence global weather changes [8,9]. 

The present trends for particulate monitoring strategies tend to monitor PM_2.5_ and PM_10_ because of the direct relationship with health effects and the prevention of natural particulate interference [10] Industrial activities with high primary particulate emissions, such as coal, cement, concrete or mining, have a significant impact on air quality due to their intensive particulate emissions in 2.5–10 µm range. The continued use of wood and coal for home heating and cooking are unavoidable issues in developing countries [11,12,13,14,15], which are also contributing to PM_2.5_ emissions.

Each country has different standards for PM_2.5_ mass concentration. To meet the standard for PM_2.5_, it is desirable to find the factors affecting PM_2.5_ concentration [16]. There are three factors that can make an important effect on the PM_2.5_ mass concentration, including domestic pollutants emission sources, external sources outside of the country, and the meteorological conditions. In Japan, the very stringent restrictions on emission sources have resulted in the low level of PM_2.5_ mass concentration. Therefore, the pollutants from long range transport play an important role on the PM_2.5_ concentration. 

As shown by previous studies, meteorological conditions can largely diffuse, dilute, and accumulate pollutants [17]; thus, PM_2.5_ mass concentration is mainly due to meteorological conditions [18]. Yang *et al*. [19] concluded that meteorological conditions can, at least, make a contribution of 16% to the reduction of PM_2.5_ mass concentration. Yerramilli *et al*. [20] integrated the Hybrid Single-Particle Lagrangian Integrated Trajectory (HYSPLIT) atmospheric dispersion model, driven by the advanced research version of Weather Research and Forecasting (WRF) mesoscale atmospheric model, to assess source location, transportation trends, and the extent of contribution to PM_2.5_.

In this paper, PM_2.5_ data and meteorological variables were collected during 1 January 2013~31 December 2013. ArcGIS10.0 was used to interpolate and plot the PM_2.5_ spatial distribution. Linear analysis and Spearman analysis were used to analyze the correlation between PM_2.5_ and meteorological variables. The relationship between PM_2.5_ mass concentration and meteorological conditions were used to deduce the external pollutant sources. Data processing was used to analyze seasonal and daily distribution of PM_2.5_.

## 2. Data and Method

### 2.1. Data in Nagasaki

**Figure 1:**
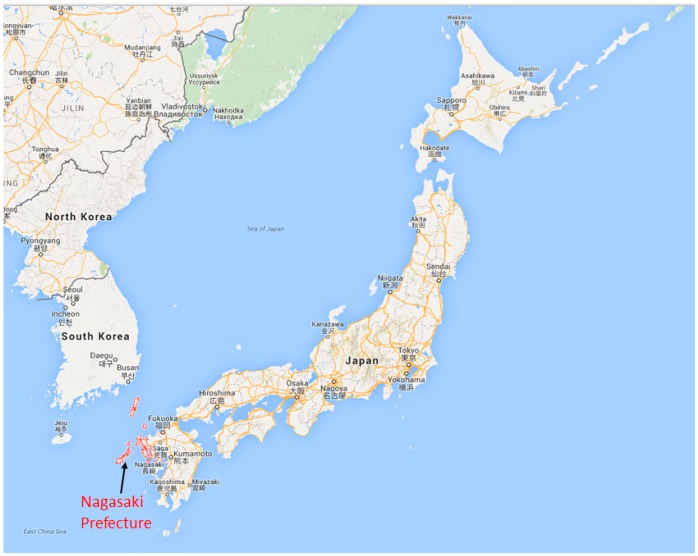
Location of Nagasaki Prefecture in Japan.

**Figure 2:**
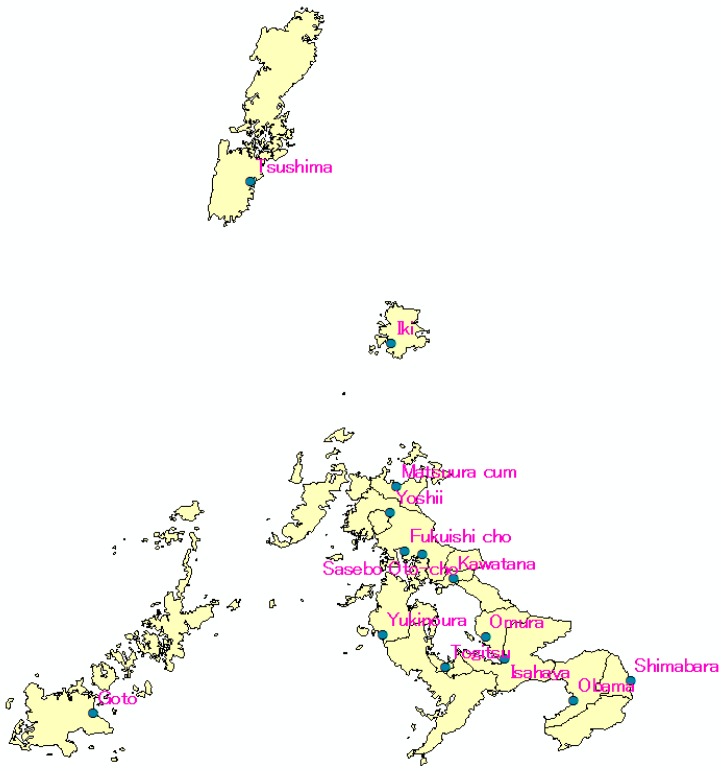
PM_2.5_ monitoring stations in Nagasaki prefecture.

The study was carried in Nagasaki Prefecture, Japan. Nagasaki is located at the westernmost part of Japan (Figure 1). It is a coastal tourist city with a small number of factories and forest coverage higher than 60%. Nagasaki’s domestic anthropogenic sources contribute much less than eternal sources from outside of Japan. This attribute of Nagasaki makes it an ideal candidate to study external sources from outside of the country by long range transport. As shown in the Figure 2, there are many monitoring stations in Nagasaki. The data that utilized for this study can be found at Nagasaki prefecture Government website (http://www.pref.nagasaki.jp/); PM_2.5_ mass concentration was collected during 1 January 2013~31 December 2013. Meteorological data of Nagasaki in 2013 (temperature, humidity, wind speed, and wind direction) were collected from the Japan Meteorological Agency website [21]. 

### 2.2. Data Processing Method 

#### 2.2.1. Spatial and Temporal Distribution 

In order to obtain accurate spatial distribution of PM_2.5_ concentrations the data of February 2014 were used. PM_2.5_ pollution is most serious in February in Nagasaki prefecture. Data processing was used to obtain the monthly values of PM_2.5_ and interpolation was conducted in ArcGIS10.0 to obtain a PM_2.5_ spatial distribution in Nagasaki prefecture. Furthermore, data processing was conducted to obtain monthly average values to derive daily and seasonal distribution. 

#### 2.2.2. Correlations between PM_2.5_ and Meteorological Variables

The PM_2.5_ data and meteorological data were collected for the whole year in 2013. Firstly, the data was divided into four seasons (spring: March, April, and May, summer: June, July, and August, autumn: September, October, and November, winter: December, January, and February). Secondly, unary linear regression and Spearman rank correlation analysis were conducted between PM_2.5_ values and meteorological variables. In previous studies unary linear regression, multiple linear regression or Spearman rank correlation analysis were used. This paper used both the unary linear regression and the Spearman rank correlation analysis. This was done in order to compare the difference between the two methods.

For meteorological variables which include temperature, humidity and wind speed, the hourly average values, for 24 h during 1 January 2013~31 December 2013, were used. This was done because PM_2.5_ values have the largest range in one day, which allowed for identifying the correlations with meteorological variables. For precipitation and wind direction, the average values per day during 1 January 2013~31 December 2013 were used. Days with precipitation ≥1 mm were selected to analyze the correlation with PM_2.5_.

SPSS is a widely used program for statistical analysis. In this paper Spearman analysis was conducted in SPSS to analyze the correlation between meteorological factors: temperature, humidity, and wind speed.

Wind direction is crucial to determine PM_2.5_ concentrations that result from pollutants by long range transport in Nagasaki. Pohjola *et al*. [17] has implied that PM_10_ concentrations originate mainly from local vehicular traffic (direct emissions and resuspension), while the PM_2.5_ concentrations are mostly of regionally- and long-range transported origin. The weighted PM_2.5_ values by wind speed were used to distinguish which direction brings the most pollutants from outside of the study area.

HYSPLIT model was designed to compute both simple air parcel trajectories and complex dispersion and deposition simulations. In this paper it was used to produce back trajectories of air parcels originating from Nagasaki. The back trajectories provide the Lagrangian path of the air parcels in the chosen time scale, which will be useful to identify the source locations of the pollutants that fall in the track of the back trajectories. 

For each direction, the weighted PM_2.5_ values were acquired using the following equation:

(1) $$\text{AWP=}\frac{\sum_{\text{i}}\frac{\text{P}}{\text{WS}}}{\text{N}}，1\leq \text{i}\leq \text{N}$$

where, AWP is the average weighted PM_2.5_ value in a year, P is the everyday PM_2.5_ value, WS is the corresponding wind speed, and N is the times which the wind direction attended in a year.

## 3. Results and Discussion

### 3.1. Spatial Distribution 

**Figure 3:**
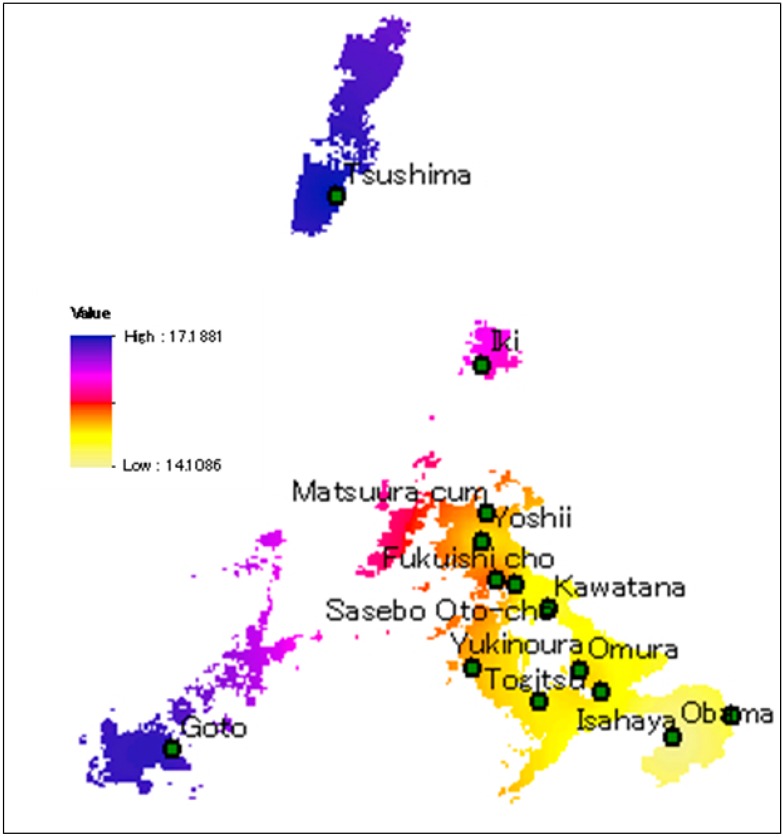
Spatial distribution of PM_2.5_ mass concentration in Nagasaki prefecture.

The seasonal distribution analysis depicted that the most polluted month in 2013 was February. Therefore, Arcgis10.0 was used to illustrate the spatial distribution of PM_2.5_ mass concentration data in February in Nagasaki (Figure 3). The westernmost part of the map depicts the most serious pollution. In the easternmost part, the pollution was least serious. The results verified that the main source of PM_2.5_ in Nagasaki is mainly from long range transport from East Asia.

### 3.2. Correlations with Meteorological Variables

**Figure 4:**
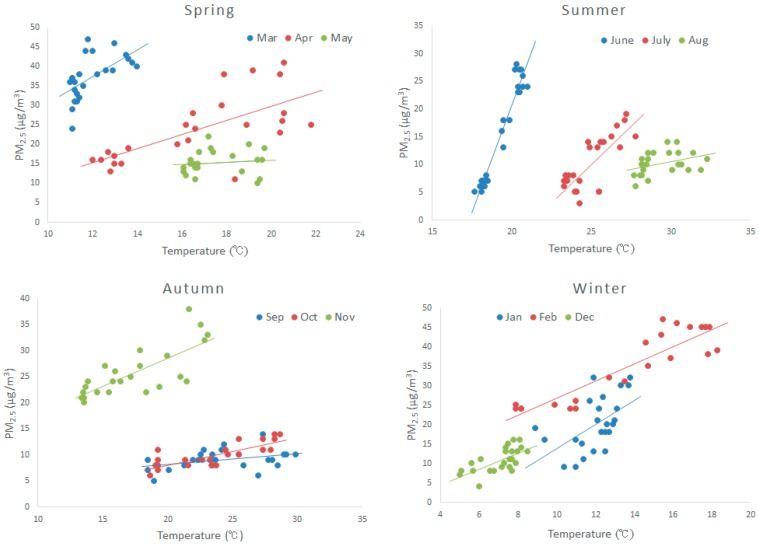
The relationship between PM_2.5_ and temperature over four seasons. The straight lines are the unary linear regression.

Figure 4 shows the relationship between PM_2.5_ and temperature. For the majority of the months PM_2.5_ has a strong positive correlation with temperature. This is because temperature can affect the formation of particles; thus, the high temperature can promote the photochemical reaction between precursors.

Table 1 depicts the R^2^ coefficient comparison between unary linear analysis and Spearman analysis. The unary linear analysis results indicate that R^2^ has the range from 0.1 to 0.9 which is in agreement with [18,22]. Furthermore, the Spearman analysis indicates that R^2^ has a range from 0.3 to 0.9. The R^2^ values resulting from Spearman analysis is higher than R^2^ using unary linear analysis (Table 1).

**Table 1:** Correlation between PM_2.5_ and temperature in a year.

R^2^	1	2	3	4	5	6	7	8	9	10	11	12
Linear analysis	0.261	0.303	0.359	0.464	0.017	0.936	0.602	0.139	0.137	0.645	0.562	0.431
Spearman analysis	0.6	0.623	0.721	0.714	0.327	0.887	0.657	0.434	0.372	0.804	0.767	0.683

**Figure 5:**
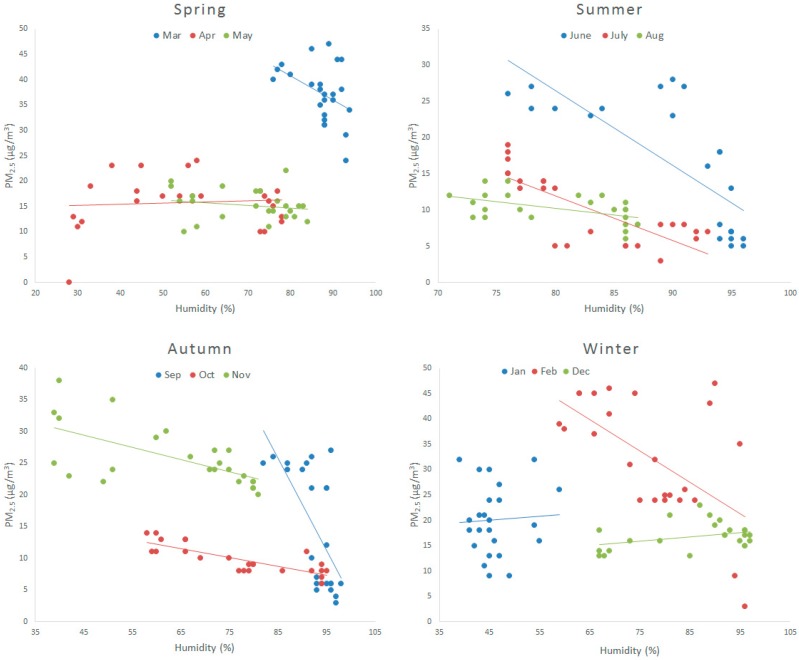
The relationship between PM_2.5_ and humidity during the year.

Figure 5 shows the linear correlation between PM_2.5_ concentration and humidity. In most of the months, PM_2.5_ has a strong negative correlation with humidity. In some months there are positive correlations but the correlation coefficient is very low. In summer, when the humidity is higher than 70%, PM_2.5_ concentration has a strong negative correlation with humidity. In autumn the correlation is all negative. In September, the humidity is very high ranging between 80%~100%. There is a strong correlation of 0.4 and 0.6, respectively, using linear analysis and Spearman analysis. PM_2.5_ concentration decreases quickly when humidity increases in the summer months and in September. In spring and winter humidity is relatively low, with a correlation coefficient lower than 0.1, except in February and March.

In February and March, the humidity is relatively high, between 60%~100% and 80%~100%, respectively. When the humidity is low, because of hygroscopic growth, PM_2.5_ concentration increases [23]. When the humidity is high enough, the particles grow too heavy to stay in the air. Therefore, dry deposition occurs; particles fall to the ground. As a result, particle numbers reduce and PM_2.5_ concentration decreases. Table 2 displays the correlation coefficients obtained by the linear analysis and Spearman analysis, respectively.

**Table 2:** Correlation between PM_2.5_ and humidity in a year.

R^2^	1	2	3	4	5	6	7	8	9	10	11	12
Linear analysis	0.003	−0.345	−0.178	0.007	−0.035	−0.577	−0.602	−0.263	−0.421	−0.657	−0.405	0.129
Spearman analysis	−0.044	−0.505	−0.4	−0.023	−0.304	−0.856	−0.749	−0.555	−0.665	−0.804	−0.704	0.224

**Figure 6:**
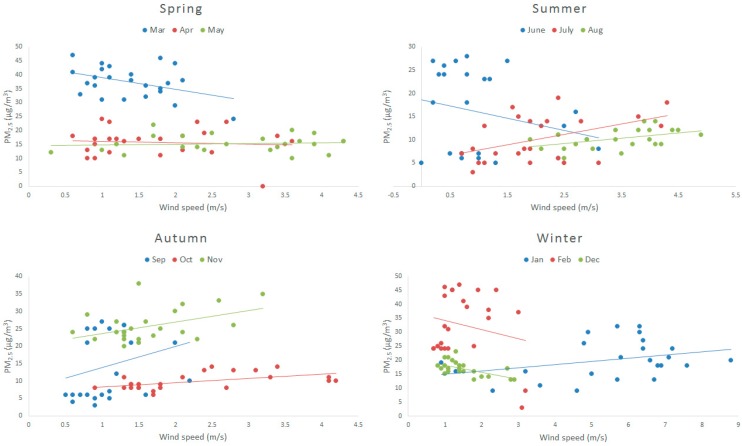
The relationship between PM_2.5_ and wind speed in a year.

Figure 6 shows negative correlation between PM_2.5_ and wind speed lower than 3 m/s and positive correlation between PM_2.5_ and wind speed higher than 3 m/s. When the wind speed is low, it can blow away the pollutants within a certain geographical range but, when the wind speed is high enough, it can transport large quantities of pollutants from far away. Table 3 show the results derived from the Spearman analysis and linear analysis. The results obtained by the Spearman analysis reflect better correlation between wind speed and PM_2.5_.

**Table 3:** Correlation between PM_2.5_ and wind speed in a year.

R^2^	1	2	3	4	5	6	7	8	9	10	11	12
Linear analysis	0.11	−0.045	−0.176	−0.007	0.008	−0.061	0.212	−0.212	0.072	0.289	0.186	−0.311
Spearman analysis	0.286	0.166	−0.287	0.124	0.153	−0.182	0.377	0.43	0.382	0.554	0.334	−0.516

**Figure 7:**
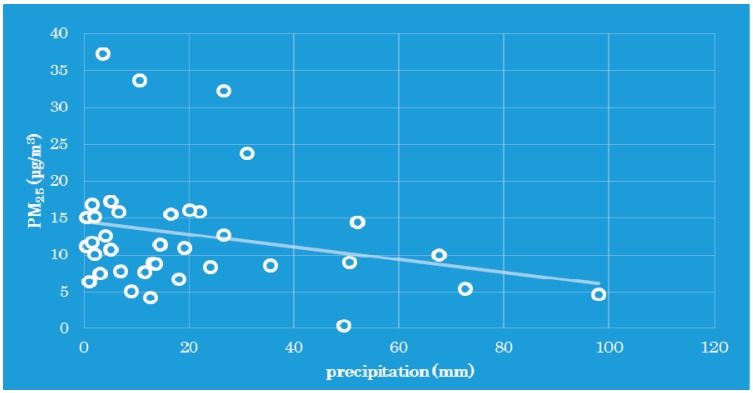
The relationship between PM_2.5_ and precipitation during the year.

Precipitation can effectively decrease PM_2.5_ mass concentrations through wet deposition (Figure 7). Precipitation can effectively remove atmospheric particulate matter, especially of small size. The linear regression and Spearman analysis indicated that the correlation between PM_2.5_ and precipitation was negative with coefficients of −0.0606 and −0.197. The minus means negative correlation, that is, the PM_2.5_ concentration decreases with increasing precipitation.

**Figure 8:**
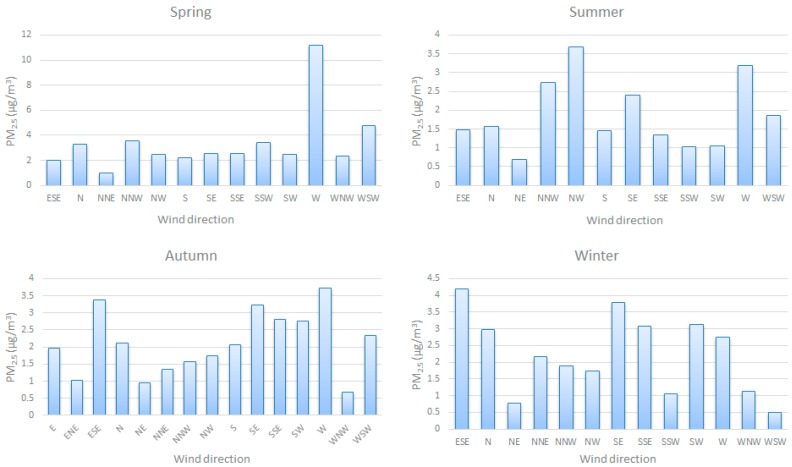
The relationship between PM_2.5_ and wind direction in a year.

The wind direction is an important parameter affecting PM_2.5_ [21]. The wind from different directions transported different amount of pollutants. Figure 8 shows the AWP (Average Weighted PM_2.5_ by wind speed) for different wind directions for the four seasons. In spring, the west wind transported the most pollutants. In summer, the NNW wind, NW wind, SE wind, and W wind transported more pollutants than wind in other directions. In autumn, the ESE wind, SE wind, and W wind transported more pollutants than wind in any other directions. In winter, the ESE wind, N wind, SE wind, SSE wind, SW wind, and W wind transported more pollutants than wind in other directions. In every season, the west wind always transported more pollutants than wind in other directions. It can be concluded that the pollutants in Nagasaki are mainly from East Asia. 

**Figure 9:**
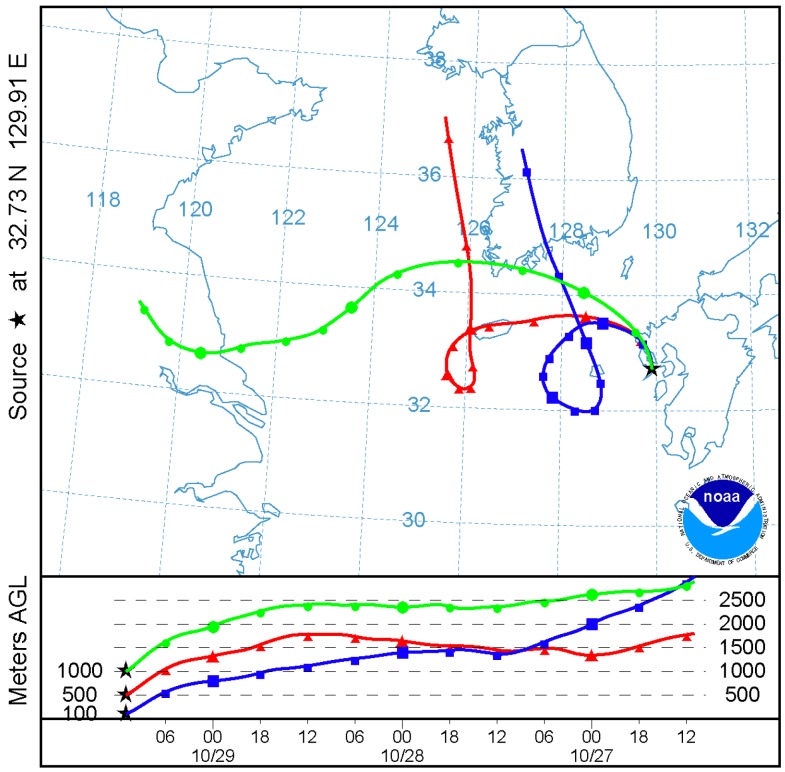
Computed back trajectories for the 72 h period ending 1100 UTC on 19 June 2009 from the GDAS Meteorological data. Upper part shows the horizontal path and the lower part shows the vertical path of the trajectories.

This can be verified by the back trajectories of air parcels obtained by the HYSPLIT model, as shown in Figure 9, which shows the sources could be East Asia. 

### 3.3. Temporal Distribution

**Figure 10:**
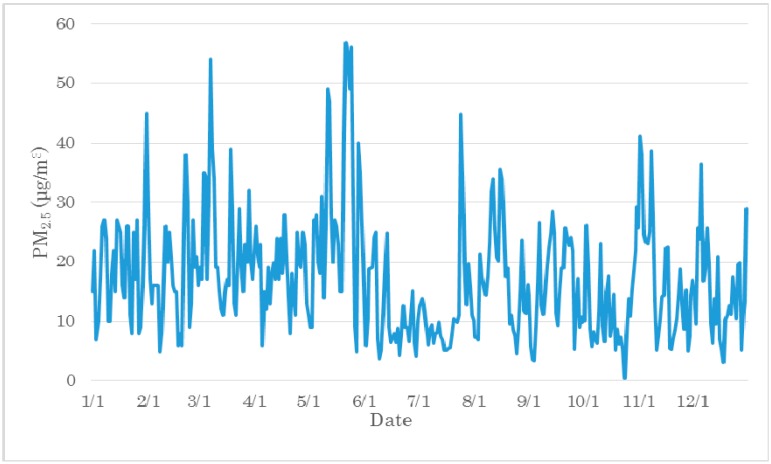
Daily PM_2.5_ mass concentration variation in Nagasaki.

From the average PM_2.5_ mass concentrations for 24 h during 1 January 2013~31 December 2013, the temporal distribution of PM_2.5_ mass concentration was obtained (Figure 10). As illustrated in Figure 10, there are differences in daily PM_2.5_ mass concentrations. The pollution mass concentration could have a larger difference in longer periods of time than in shorter time periods. In this case, the source concentrations and the temperature conditions affect the final PM_2.5_ mass concentrations. As shown in Figure 10, around 1 February, in a short period, the pollution mass concentrations has a larger difference. In such situations the meteorological conditions, such as wind direction, speed, and precipitation, could play an important role in increasing or decreasing the mass concentrations. 

**Figure 11:**
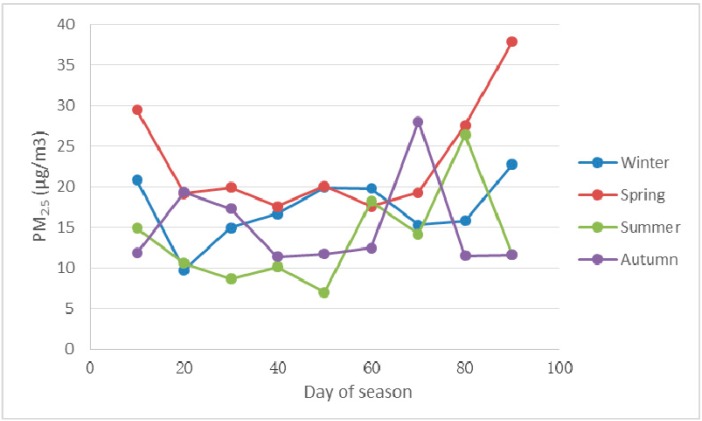
Seasonal PM_2.5_ mass concentration variation in Nagasaki.

From the data collected, the average values for every 10 days were calculated in order to estimate the seasonal PM_2.5_ mass concentration distribution (Figure 11). The results show that there is a decrease of PM_2.5_ pollution mass concentrations in the following order: spring, autumn, winter, summer. The decreasing order differs from the China mainland and Taiwan, where the order is as follow: winter, spring, autumn, summer. In spring the long range transport affected by meteorological conditions, especially wind direction, results in higher concentrations. In summer, the most important factor is precipitation, which effectively decreases pollutant concentration. In addition, the high temperature, to some extent, hindered the formation of particles. During the winter, in China, the high PM_2.5_ concentrations are mostly caused by the combination of a strong domestic emission. The combustion of crop straw for heating in rural areas and the low mixing heights suppress the diffusion of pollutants. However, in Nagasaki, during the winter, there is no strong local emission and due to the meteorological conditions, especially wind direction, the long range transport is weakened.

## 4. Conclusions

In this paper, PM_2.5_ data and meteorological variables were collected in Nagasaki during 1 January 2013~31 December 2013. Through interpolation in ArcGIS10.0, the PM_2.5_ spatial distribution for Nagasaki prefecture was obtained. As the map depicts, the westernmost part of Nagasaki prefecture displayed the most serious pollution. The easternmost part displays less serious pollution. The results reinforce the idea that the main source of PM_2.5_, in Nagasaki, is from East Asia. The seasonal distribution and daily distribution were analyzed utilizing data processing. Linear analysis and Spearman analysis were utilized to find the correlation between PM_2.5_ mass concentrations and meteorological variables. The results showed that precipitation had a negative correlation with PM_2.5_ and temperature was positively correlated to PM_2.5_; the correlations between PM_2.5_ and wind speed and humidity had a threshold and the west wind transports the most pollutants to Nagasaki in all four seasons. It can be concluded that the pollutants in Nagasaki may mainly from East Asia through long range transport.
